# Prevalence and drug susceptibility pattern of group B *Streptococci* (GBS) among pregnant women attending antenatal care (ANC) in Nekemte Referral Hospital (NRH), Nekemte, Ethiopia

**DOI:** 10.1186/s13104-017-2725-3

**Published:** 2017-08-10

**Authors:** Hylemariam Mihiretie Mengist, Olifan Zewdie, Adugna Belew, Regea Dabsu

**Affiliations:** Department of Medical Laboratory Sciences, College of Medical and Health Sciences, Wollega University, P.O. Box: 395, Nekemte, Ethiopia

**Keywords:** Prevalence of GBS, Drug susceptibility pattern, Pregnant women, Nekemte

## Abstract

**Objective:**

The main objective of this study was to determine the prevalence and drug susceptibility pattern of group B *Streptococci* (GBS) among pregnant women. The specific objectives include; (1) To determine the prevalence of *GBS* colonization among pregnant women (2) To determine the drug susceptibility pattern of *GBS* among pregnant women and (3) To identify associated risk factors with *GBS* colonization among pregnant women.

**Results:**

The median age of the participants was 24.5 years (range 16–38) and 86% participants were urban residents. The total prevalence of maternal GBS colonization from vaginal swab culture was 12.2% (22/180). The prevalence of GBS colonization rate was significantly higher in those pregnant women above 37 weeks of gestation [AOR, 95% CI 2.1 (1.2, 11.6), P = 0.03] and married ones [AOR, 95% CI 3.2 (1.8, 11.6), P < 0.021]. Twenty (91%) of GBS isolates were sensitive to vancomycin and the highest resistance was observed against penicillin G (77.3%). The prevalence of GBS colonization in this study was significantly high and differed by gestational age and marital status. None of the GBS isolates were resistant to vancomycin but higher resistance was shown against Penicillin G.

## Introduction

Group B *Streptococcus* causes invasive disease primarily in infants, pregnant or postpartum women, and older adults, with the highest incidence among young infants. Maternal infections of GBS constitute one of the leading pathogens associated with both early and late-onset neonatal sepsis. Maternal vaginal GBS colonization is related with early onset of neonatal sepsis and subsequent colonization during birth occurs in approximately 70–75% of infants [1–4].

Studies state that maternal intrapartum GBS colonization is the primary risk factor for early-onset disease in infants. A study conducted in the 1980s revealed that pregnant women with GBS colonization were >25 times more likely than pregnant women with negative prenatal cultures to deliver infants with early-onset GBS disease [5].

Although intrapartum prophylaxis has been established to lead to a 70% decline in the incidence of GBS disease, early-onset GBS disease remains a leading cause of illness and death among newborns less than the age of 7 days [6]. GBS colonization rate is significantly important and the isolates are variably sensitive to different antimicrobials as revealed by different studies from Ethiopia [7, 8].

There is a paucity of published data concerning maternal GBS colonization and antimicrobial susceptibility patterns in West Ethiopia particularly in the study area (Nekemte). The present study is, therefore, aimed to determine prevalence and drug susceptibility pattern of GBS among pregnant women attending antenatal care (ANC) in Nekemte Town, Western Ethiopia.

## Main text

### Materials and methods

#### Study setting and context

A prospective cross-sectional study was conducted between March and May 2016 in Nekemte Referral Hospital (NRH).

#### Sample size and sampling technique

Sample size was calculated using single population proportion formula considering 95% CI and P = 0.21 from the previous study conducted in Hawassa, Ethiopia [8] as follows;

$${\text{n}} = {\text{Z}}_{{({\text{a}}/ 2)}}^{ 2} {\text{P}}\left( { 1- {\text{P}}} \right)/{\text{d}}^{ 2}$$

where the average number of pregnant women attending ANC in NRH per 2 months (N) was 480. Therefore, based on correction formula (n = n_0_/(1 + n_0_/N)) and 20% contingency, a total of 180 pregnant women were enrolled consecutively in the study.

#### Study population and data collection

Pregnant women with gestational age of ≥35 weeks were included. Site assessment and pre-test were done prior to data collection and questionnaires were used to obtain data on socio-demographic, obstetric and clinical factors. The questionnaires were developed in English and participants were interviewed using local language; Afan Oromo. Nurses who can speak the local language were trained on data collection for this particular study. The data collectors were regularly supervised by the investigators.

#### Specimen collection and transportation

Vaginal swabs were collected by brushing the lower vagina with a sterile cotton swab by trained nurses following universal precautions [9]. The swabs were immediately inoculated in 1.5 ml Todd Hewitt broth (supplemented with colistin and nalidixic acid) and transported to the Medical Microbiology laboratory of Nekemte Regional Laboratory (NRL).

#### Culture and identification of GBS

The broth was incubated for 18–24 h at 35–37 °C and subcultured on 5% sheep blood agar (OXOID, UK) and then incubated overnight in 5% CO_2_ atmosphere for 18–24 h. All suspected GBS colonies (with narrow beta-hemolysis) were sub-cultured on nutrient agar and subjected to gram stain and catalase test. All gram-positive and catalase negative isolates were tested for Bacitracin sensitivity and CAMP test for confirmation.

#### CAMP test

The CAMP test is used to identify beta-hemolytic streptococci; *Streptococcus agalactiae (*group B) (CAMP positive) from *Streptococcus pyogenes* (group A) (CAMP negative). In brief, *Staphylococcus aureus* is inoculated onto a sheep blood agar plate by making a narrow streak down the center of the plate with a loop. The test organism (group B *Streptococcus*) is then streaked in a straight-line inoculum at right angles to the *S. aureus* within 2 mm far. The plates are incubated at 35 °C for 24 h. A positive CAMP result is indicated by an “arrowhead”-shaped enhanced zone of beta-hemolysis in the area between the two cultures with the “arrow point” toward the *S. aureus* streak. No enhanced zone of beta-hemolysis is observed in a CAMP negative reaction [10].

#### Antimicrobial susceptibility testing

The inoculums were prepared by suspending 4–5 isolated colonies of the same morphology in 5 ml of sterile physiological saline equal to a 0.5 McFarland’s standard used as a reference to adjust the turbidity of bacterial suspensions. Colonies were inoculated on Mueller–Hinton agar (MHA) plates supplemented with 5% defibrinated sheep blood. Then antibiotic disks were placed and incubated at 35–37 °C under 5% CO_2_ atmosphere for 20 h. Six antibiotic disks (Oxoid) namely; Penicillin G (P) (10 IU), erythromycin (E) (15 μg), ceftriaxone (CRO) (30 μg), chloramphenicol (C) (30 μg), vancomycin (VA) (30 μg) and Clindamycin (CLI) (10 μg) were used Each isolate was classified as susceptible, intermediate or resistant to each antibiotic tested based on clinical laboratory standards institute (CLSI) guideline [11].

#### Data processing and analysis

Data were cleaned, coded and double entered into EpiData 3.1 version software. Then data were exported to SPSS 24 version software for analysis. Binary logistic regression models were used to determine the association between predictors and dependent variables. P values ≤0.05 were taken to a significant level.

#### Quality control

Standard operating procedures (SOPs) were followed during sample collection, transportation, and processing steps. The proficiency of Todd–Hewitt broth was checked by inoculating the broth with known Gram-negative bacteria (*Escherichia coli*) and GBS isolates to see if it can really inhibit Gram-negative bacteria and allow growth of Gram-positive bacteria. Before using any reagents and culture media, any physical change was assessed and expiration date checked. The quality of the culture media and antimicrobial disks was checked using standard American Type Culture Collection (ATCC) reference strain of *Staphylococcus aureus* ATCC 25923, *Escherichia coli* (ATCC 25922) and *S. agalactiae* isolates (ATCC 12386).

### Results

#### Socio-demographic characteristics

A total of 180 pregnant women were recruited with a median age of 24.5 years (Range 16–38 years). As shown in Table 1, most participants were aged below 29 years old (78.3%), urban residents (86.1%), housewives (64.4%), married (95.6%) and literates (90.6%).

**Table 1:** Socio-demographic, obstetric and clinical characteristics and factors affecting maternal GBS colonization rate among pregnant women attending ANC in NRH from March to May 2016, Nekemte, Ethiopia

Variables	GBS		Total, n (%)	COR (95% CI)	AOR (95% CI)
	Positive, n (%)	Negative, n (%)			
Age group (in years)					
<29	15 (10.6)	126 (89.4)	131 (78.3)	1.4 (0.2, 12.3)	
≥29	7 (18)	32 (82)	39 (21.7)	1	
Residence					
Rural	5 (20)	20 (80)	25 (13.9)	1	
Urban	17 (11)	138 (89)	155 (86.1)	1.3 (0.8, 3.1)	
Occupation					
Farmer	3 (43)	4 (57)	7 (3.9)	–	
Housewife	12 (10.3)	104 (89.7)	116 (64.4)	3.7 (0.4, 37)	
Merchant	2 (40)	3 (60)	5 (2.8)		
Civil servant	5 (9.6)	47 (90.4)	52 (28.9)	1	
Marital status					
Single	3 (37.5)	5 (62.5)	8 (4.4)	4.7 (0.5, 46)*	3.2 (1.8, 11.6)**
Married	19 (11)	153 (89)	172 (95.6)	1	1
Educational status					
Illiterate	9 (53)	8 (47)	17 (9.4)	1	
Literate	13 (8)	150 (92)	163 (90.6)	1 (1.01, 1,08)	
Gravida					
Multigravida	14 (13.5)	90 (86.5)	104 (57.8)	1	
Primigravida	8 (10.5)	68 (89.5)	76 (42.2)	1.5 (0.3, 8.3)	
Gestational age (weeks)					
35–37	4 (3.1)	127 (96.9)	131 (72.8)	0.4 (0.07, 1.8)*	2.1 (1.2, 11.6)**
>37	18 (35.3)	31 (63.9)	49 (27.2)	1	1
Recent antibiotic treatment					
Yes	9 (17.6)	42 (82.4)	51 (28.3)	1	
No	13 (10)	116 (90)	129 (71.7)	1.3 (0.2, 7.2)	
Contraception					
Yes	10 (8)	116 (92)	126 (70)	0.4 (0.08, 2.1)	
No	12 (22.2)	42 (77.8)	54 (30)	1	

*COR* crude odds ratio, *AOR* adjusted odds ratio, *NRH* Nekemte referral hospital

* (marginally significant at P < 0.1)

** (significant at P < 0.05)

#### Obstetric and clinical characteristics

Regarding obstetric and clinical characteristics of the study participants; 104 (57.8%) were multigravida, 131 (72.8%) were between 35 and 37 weeks of gestational age, 129 (71.7%) had no history of recent antibiotic treatment and 175 (97.2%) had no history of stillbirth (Table 1).

#### Maternal GBS colonization and associated risk factors

The total GBS colonization rate from vaginal swab culture was 12.2% (22/180). From the total GBS isolates majority were from pregnant women aged below 29 years old (15, 68.2%), urban residents (17/22, 77.3%), housewives (12, 854.5%), married ones (19, 86.4%), literates (13, 59%), multigravida (14, 63.6%), with no recent antibiotic treatment (13, 59%), with no history of oral contraception use (12, 54.5%) and in those with gestational age above 37 weeks (18, 81.8%) (Table 1).

The prevalence of GBS colonization was significantly higher in those pregnant women above 37 weeks of gestation [AOR, 95% CI 2.1 (1.2, 11.6), P = 0.03] and married pregnant women [AOR, 95% CI 3.2 (1.8, 11.6), P = 0.021]. Therefore, married pregnant women and those above 37 weeks of gestation were 3.2 and 2.1 times more likely to be colonized by GBS than their counterparts, respectively (Table 1).

#### Antibiotic susceptibility pattern of GBS isolates

Twenty (91%) of GBS isolates were sensitive to vancomycin. All isolates were multi-drug resistant. The highest resistance was observed against penicillin G, 17 (77.3%). Nine (41%) of the isolates were intermediate against ceftriaxone. Half of the isolates were sensitive to clindamycin and 36.4% of them were intermediate against erythromycin (Table 2).

**Table 2:** GBS in vitro antimicrobial susceptibility pattern, 2016, Nekemte, Ethiopia

Antimicrobial	Sensitive		Intermediate		Resistant	
	Frequency (n = 22)	Percent (%)	Frequency (n = 22)	Percent (%)	Frequency (n = 22)	Percent (%)
Penicillin G (P)	3	13.6	2	9.1	17	77.3
Erythromycin (E)	9	41	8	36.4	5	22.6
Ceftriaxone (CRO)	6	27.2	9	41	7	31.8
Chloramphenicol (C)	11	50	–	–	11	50
Vancomycin (VA)	20	91	2	9	–	–
Clindamycin (CLI)	11	50	7	31.8	4	18.2

### Discussion

The prevalence of GBS colonization during pregnancy is variable [12] and different studies have reported variable susceptibility of GBS against commonly prescribed drugs that indicates the importance of performing drug susceptibility test prior to prescription.

In this study, the total GBS colonization rate from vaginal swab culture was 12.2% (22/180). Although we have assessed the association of socio-demographic, obstetric and clinical variables with GBS colonization, only gestational age, and marital status were significant predictors of maternal GBS colonization (P < 0.05). We found that most (91%) GBS isolates were sensitive to vancomycin and the highest resistance of GBS isolates was observed against penicillin G (77.3%).

This 12.2% prevalence of maternal GBS colonization in this study is comparatively lower than studies from Hawassa (20.9%) [8], Italy (17.9%) [13], the Netherlands (21%) [14], Tanzania (23%) [16], China (27.7%) [20] and Uganda (28.8%) [22]. But it is higher or comparable with studies from Hong-Kong (10.4%) [15], Nigeria (11.3, 18%) [17, 18], Argentina (7.6%) [19], Saudi (13.4%) [21], different districts of Ethiopia (Gondar, Mekelle, Adigrat and Addis Ababa) (9, 13.7, 11.3 and 7.2%) [12, 23, 24 and 25]. This result is definitely higher than studies from Mozambique (1.8%) [26] and India (2.3%) [27].

The possible reasons for the above differences might be due to differences in geography (for data outside Ethiopia), differences in sample size (smaller or higher than others), specimen differences (most studies used anovaginal swab while only vaginal swab was used in the current study which may render our results), differences in gestational age (some studies recruited pregnant women of all gestational ages, others involved gestational ages of 35–37 weeks but pregnant women with gestational ages of ≥35 weeks were recruited the current study), and differences in sampling frame (most studies included both health center and hospital ANC attendants unlike the current study which was based at referral hospital which in turn may decrease GBS colonization rates).

Only gestational age above 37 weeks and being married were significant predictors of GBS colonization in the current study. This is consistent with different studies (8, 16, 18, 22 and 23) where authors reported a few significant risk factors for the higher maternal GBS colonization rates.

Regarding the antimicrobial susceptibility pattern of GBS isolates, most of GBS isolates were sensitive to vancomycin while the highest resistance was observed against penicillin and variable sensitivity to clindamycin and erythromycin. Except for vancomycin sensitivity, resistance of GBS isolates against penicillin G in the present study is in contrary from a study done in Saudi [21] where all isolates were sensitive to penicillin G. This high rate of GBS resistance in the current study might be due to differences in laboratory procedures and differences in community awareness in avoiding using non-prescribed Penicillin. The drug susceptibility pattern of GBS isolates to different antimicrobial discs in this study was partly consistent with different studies from abroad [18, 19, 27, 28] and from Ethiopia [8, 23–25].

Two or more GBS isolates were intermediately sensitive to penicillin, erythromycin, ceftriaxone, and clindamycin. This is partly consistent with data from Mekelle and Adigrat [23, 24] but different from Hawassa and Addis Ababa [8, 25]. Three (13.6%) GBS isolates were sensitive to penicillin G in this study which is different from another finding in different districts of Ethiopia; Mekelle, Adigrat and Addis Ababa [23–25] where authors reported a higher sensitivity of GBS to Penicillin. This may be due to the wide and non-prescription use of penicillin because of weak drug control mechanism in different settings of the country.

The differences in antimicrobial susceptibility patterns of GBS isolates against different antimicrobials might be due to the occurrence of differences regarding bacterial strain, laboratory procedures, bacterial load, laboratory facility, drug control policies and awareness of the community towards drug resistance. The findings of this research may help clinicians and policy makers to understand the magnitude of the problem and advance the fight against empirical treatment to prevent drug resistance.

### Conclusion

The prevalence of maternal GBS colonization in this study is significantly high. Older gestational age and being married significantly increased the rate of maternal GBS colonization. None of the GBS isolates were resistant to vancomycin and half of the isolates were sensitive to chloramphenicol. Increased resistance was observed against Penicillin G and growing resistance was observed against erythromycin, clindamycin, and ceftriaxone. Screening of pregnant women for GBS colonization, AST before prescription, large-scale epidemiological studies are recommended in Ethiopia which will aid to put into practice antibiotics prophylaxis.

## Limitations

This study has main limitations in terms of small sample size, non-probability sampling method, using disc diffusion for AST, exclusion of rectal swab and exclusion of neonates.

## Abbreviations

AST: antimicrobial susceptibility test
ANC: antenatal care
AOR: adjusted odds ratio
CLSI: clinical laboratory standards institute
COR: crude odds ratio
GBS: group B *Streptococci*
NRH: nekemte referral hospital
NRL: nekemte regional laboratory

## References

[1] Phares CR, Lynfield R, Farley MM, Mohle-Boetani J, Harrison LH, Petit S (2008). Epidemiology of invasive group B streptococcal disease in the United States, 1999–2005. JAMA.

[2] Apgar BS, Greenberg G, Yen G (2005). Prevention of group B streptococcal disease in the newborn. Am Fam Physician.

[3] Goodman JR, Berg RL, Gribble RK, Meier PR, Fee SC, Mitchell PD (1997). A longitudinal study of group B streptococcus carriage in pregnancy. Infect Dis Obstet Gynecol..

[4] Boyer KM, Gadzala CA, Burd LI, Fisher DE, Paton JB, Gotoff SP (1983). Selective intrapartum chemoprophylaxis of neonatal group B streptococcal early-onset disease. I. Epidemiologic rationale. J Infect Dis.

[5] Boyer KM, Gotoff SP (1985). Strategies for chemoprophylaxis of GBS early-onset infections. Antibiot Chemother.

[6] Benitz WE, Gould JB, Druzin ML (2000). Antimicrobial prevention of early-onset group B streptococcal sepsis: estimates of risk reduction based on a critical literature review. Pediatrics.

[7] Schmidt J, Halle E, Halle H, Mohammed T, Gunther E (1989). Colonization of pregnant women and their newborn infants with group B *Streptococci* in the Gondar College of Medical Sciences. Ethiop Med J.

[8] Muhammed M, Asrat D, Woldeamanuel Y, Demissie A (2012). Prevalence of group B *Streptococcus* colonization among pregnant women attending antenatal clinic of Hawassa Health Center, Hawassa, Ethiopia. Ethiop J Health Dev.

[9] Association of Clinical Microbiologists (2006). Processing swabs for group B Streptococcal carriage. Health Prot Agency.

[10] Rahbar M, Hajia M, Mohammadzadeh M (2012). Urinary tract infections caused by GBS in adult women: survey of 11,800 urine culture results. Iran J Pathol..

[11] Wayne PA (2013). Performance standards for antimicrobial susceptibility testing. CLSI approved standard M100–S23. Clin Lab Stand Inst..

[12] Shet A, Ferrieri P (2004). Neonatal & maternal group B *Streptococcal* infections: a comprehensive review. Indian J Med Res.

[13] Busetti M, D’Agaro P, Campello C (2007). Group B *Streptococcus* prevalence in pregnant women from North-Eastern Italy: advantage of screening strategy based on direct plating plus broth enrichment. J Clin Pathol.

[14] Valkenburg-van den Berg AW, Sprij AJ, Oostvogel PM, Mutsaers JA, Renes WB, Rosendaal FR (2006). Prevalence of colonization with group B *Streptococci* in pregnant women of a multi-ethnic population in The Netherlands. Eur J Obstet Gynecol Reprod Biol.

[15] Tsui HY, Ip M, Ng P, Sahota DS, Leung T, Lau T (2009). Change in prevalence of group B *Streptococcus* maternal colonization in Hong Kong. Hong Kong Med J.

[16] Joachim A, Matee I, Massawe F, Lyamuya E (2009). Maternal and neonatal colonization of group B *streptococcus* at Muhimbili National Hospital in Dar es Salaam, Tanzania: prevalence, risk factors and antimicrobial resistance. BMC Public Health.

[17] Onipede A, Adefusi O, Adeyemi A, Adejuyigbe E, Oyelese A, Ogunniyi T (2012). Group B *Streptococcus* carriage during late pregnancy in Ile-Ife, Nigeria. Afr J Clin Exper Microbiol.

[18] Ezeonu IM, Agbo MC (2014). Incidence and antimicrobial resistance profile of group B *Streptococcus* (GBS) infection in pregnant women in Nsukka, Enugu State, Nigeria. Afr J Microbiol Res.

[19] Quiroga M, Pegeles E, Oviedo P, Pereyra E, Vergara M (2008). antibiotic susceptibility patterns and prevalence of group B *Streptococcus* isolated from pregnant women in Misiones, Argentina. Braz J Microbiol.

[20] Bidgani S, Navidifar T, Najafian M, Amin M (2016). Comparison of group B Streptococci colonization in vaginal and rectal specimens by culture method and polymerase chain reaction technique. J Chin Med Assoc.

[21] Khan M, Faiz A, Ashshi A (2015). Maternal colonization of group B *Streptococcus*: prevalence, associated factors, and antimicrobial resistance. Ann Saudi Med.

[22] Namugongo A, Bazira J, Fajardot Y, Joseph N (2016). Group B *Streptococcus* colonization among pregnant women attending antenatal care at tertiary hospital in Rural Southwestern Uganda. Int J Microbiol.

[23] Alemseged G, Niguse S, Hailekiros H, Abdulkadir M, Saravanan M, Asmelash T (2015). Isolation and antimicrobial susceptibility pattern of group B *Streptococcus* among pregnant women attending antenatal clinics in Ayder Referral Hospital and Mekelle Health Center, Mekelle, Northern Ethiopia. BMC Res Notes.

[24] Gebremesker TK, Zeleke TA, Mihiret A, Tikue MD (2015). Prevalence and antibiotic susceptibility Pattern of *Streptococcus agalactiae* among pregnant women at Adigrat Zonal Hospital and Adigrat Health Center, Tigray, Ethiopia. SPG J Gynecol Obstet.

[25] Woldu ZL, Teklehaimanot TG, Waji ST, Gebremariam MY (2014). The prevalence of Group B Streptococcus recto-vaginal colonization and antimicrobial susceptibility pattern in pregnant mothers at two hospitals of Addis Ababa, Ethiopia. BMC Reprod Health.

[26] De Steenwinkel DO, Tak HU, Muller AE, Nouwen JL, Oostvogel PM, Mocumbi SM (2008). Low carriage rate of group B *Streptococcus* in pregnant women in Maputo, Mozambique. Trop Med Int health.

[27] Sharmila V, Joseph N, Babu T, Latha C, Sistla S (2011). Genital tract group B streptococcal colonization in pregnant women: a South Indian perspective. Infect Dev Ctries.

[28] Strus M, Pawlik D, Wloch M, Gosiewosky T, Heczko B, Laurebach R (2008). Group B streptococcus colonization of pregnant women and their children observed on obstetric and neonatal wards of the University Hospital in Krakow, Poland. J Microbiol.

